# Consumer Preference for Nutritionally Fortified Eggs and Impact of Health Benefit Information

**DOI:** 10.3390/foods11081145

**Published:** 2022-04-15

**Authors:** Yixing Tian, Hong Zhu, Lei Zhang, Honghua Chen

**Affiliations:** 1 College of Economics and Management, China Agricultural University, #17 Qinghua East Road, Haidian District, Beijing 100083, China; yixing_tian@cau.edu.cn (Y.T.); hongyang603@126.com (L.Z.); 2 Institute of Food and Nutrition Development, Ministry of Agriculture and Rural Affairs of the People’s Republic of China, #12 Zhong Guan Cun Southern Street, Haidian District, Beijing 100081, China; zhuhong@caas.cn

**Keywords:** functional food, information treatment, discrete choice experiment, consumer preference, willingness to pay, egg attributes

## Abstract

The potential contribution of nutritionally fortified foods to the improvement of public health has been recognized internationally; however, the extent of people’s preferences for functional foods and the influence of information intervention on consumers’ acceptance and selection of nutritious foods have not been comprehensively studied in China. The main purposes of this study are to assess Chinese consumers’ perceptions towards nutritionally fortified eggs and to explore the ways in which information about the health benefits and the international market status quo of functional eggs impacts Chinese consumers’ preferences and their willingness to pay (WTP) for nutritional fortification. Discrete choice experiments were used to elicit the preferences of 740 egg consumers from four cities in China, and a mixed logit model subsequently utilized to interpret the results. It was found that the provision of comprehensive information regarding the health benefits of trace elements and unsaturated fatty acids, as well as insight into the current market status quo, significantly improved participants’ preferences and their WTP for functional eggs. Furthermore, the heterogeneous effects of demographic and sociocultural factors on consumers’ treatment of this information were explored. It was found that the study participants with children and those with prior purchase experience exhibited a relatively stronger response to the information, while those who had expressed trust in the human health benefits of the nutritional content of functional eggs were not as sensitive as expected to the additional information. Therefore, if the government and enterprises design appropriate information treatment and nudging methods according to the current consumption characteristics of nutritionally fortified eggs, this will help to improve consumers’ purchase confidence in the health efficacy of functional food and play a positive role in promoting people’s healthy food consumption.

## 1. Introduction

Functional foods have been shown to enhance the quality of the human diet, decrease the potential risks of some chronic diseases, and effectively improve public health at a relatively low cost, thereby contributing to existing health interventions [1,2]. Hence, the practice of improving nutritional imbalance through food fortification is widely and globally accepted [3]. In particular, eggs are of interest in terms of functionality [1,4] and nutritionally fortified eggs, also called nutrition-enriched or functional eggs, are among those products experiencing rapid growth in recent years worldwide [3,5]. Producers improve the nutritional quality of eggs by enhancing the nutrients in poultry feed. Nutritionally enriched products currently include selenium-enriched eggs, folic acid-enriched eggs, and unsaturated fatty acid-enriched eggs [6].

While there is significant potential demand amongst Chinese consumers for foods of nutritional quality that can contribute to dietary health, this is not currently reflected in the sales and purchases of nutritionally fortified products [7,8]. This may be due to insufficient relevant nutritional and health information about the emerging niche products, such as nutritionally fortified eggs, together with minimal previous purchase experience; hence, the Chinese market is still underdeveloped compared with those of developed countries. Consumers tend to adopt a conservative, risk-averse attitude when presented with new products that are not supported by sufficient data to enable their purchase decisions; however, they are simultaneously sensitive to any additional information about such products [9,10]. Information plays an essential role in shaping consumer perceptions of innovated products by creating awareness, imparting knowledge, and forming or changing an individual’s existing cognition and attitude [11,12].

Some studies have explored the ways in which food composition tables and nutrition claims as an external information treatment affecting purchasing decisions about functional foods, and found that they help consumers to make more informed food choices in their daily lives [13,14,15,16,17]. However, labels do not always have a significant impact on all consumers, especially when consumers are poorly informed about functional foods, including nutritionally enhanced eggs, and do not recognize the values of the food composition tables on functional foods packaging [18,19,20,21]. The findings of Ahn*,* et al. [22], Pasquale*,* et al. [23], and Markosyan, Mccluskey and Wahl [10] imply that more objective information regarding the concept of functional foods and their potential health benefits can have a positive significant effect on consumers’ WTP. Thus, a comprehensive health claim about functional eggs may be beneficial to the relevant stakeholders and policymakers, and support the promotion of consumer awareness amongst those not fully aware of the health benefits [8].

One interesting and pertinent question, however, is: how do Chinese consumers respond to external or Supplementary Information regarding nutrition-enhanced eggs? To date, the impacts of such information on the perceptions and acceptance of functional foods have not been extensively studied in China, and the ways in which Chinese consumers are attracted and stimulated to purchase functional food is far from being understood. To a certain extent, developing countries are facing common problems in the development of dietary nutrition education and the promotion of nutritionally fortified foods. There may be similar or consistent responses in many other regions or countries to how information intervention affects consumer choice. Along with the rapid development of functional food on the international market, China is considered one of its most promising markets globally. Studying Chinese consumers’ preference for functional food and their response to information treatment will provide a research basis for the follow-up comparison with developed countries. This study will also help to further explore the reasons for the differences between different countries.

Although some studies show that information plays an essential role in shaping consumer perceptions of innovated products by creating awareness, imparting knowledge, and forming or changing an individual’s existing cognition and attitude [15,24], the existing literature is still insufficient and further research is needed due to the perceived deficiency in the following aspects. First, little attention is paid to the impact of information treatment on consumers’ choice preference for food nutritional quality, which hinders understanding of market segments and the formulation of marketing campaigns. Moreover, the relevant functional food stakeholders in China cannot simply utilize the findings from other countries and regions, and thus require specific Chinese consumer research to be widely conducted. Second, at present, the content of information intervention is relatively simple. Few studies consider whether consumers will refer to other people’s behavior of purchasing nutritious eggs so as to change their own choice preference. Third, the consumer group characteristics of potential nutritional fortification preference are not clear, so it is impossible to form a comprehensive and objective judgment on the actual effect of information treatment. There are two previous research studies that bear a slight resemblance to this present study. Żakowska-Biemans and Tekień [5] investigated Polish consumer perceptions of information regarding the farming system (including organic and free-range) and nutritional enhancement of eggs, to assess their acceptance of claims combining both sustainability and nutrition-related health benefits. Yeh*,* et al. [25] extended the understanding of trade-offs between ‘organic’ labels and other nutrition claims, consequently identifying consumer segments with similar preferences in Italy and Hungary. Nevertheless, neither of these two studies included both organic/free-range and the nutritional enhancement of eggs as attributes in affecting the overall premium for eggs, nor did they investigate whether nutritional fortification is substitute or completed with other attributes under the information treatment.

Improving residents’ health through diet has become an important area of focus and a formal policy goal around the whole world. The launch of novel nutritionally enhanced eggs not only provides potential benefits for consumers’ diets, but increases new business opportunities for producers and farmers. Nutritionally enriched eggs are very common in supermarkets across the United States, Canada, and the European Union, which have formed widely accepted standards systems for functional eggs [26]. In China, however, functional foods are still an emerging, although highly anticipated, industry. In 2021, the Chinese government revised the national standard (GB 28050) on claims of unsaturated fatty acids enrichment in food products. In January 2022, China’s Ministry of Agriculture and Rural Areas issued the following two national industry standards for nutritionally fortified eggs: omega-3 polyunsaturated fatty acid fortified eggs (NY/T 4069-2021), and the omega-3 technical specifications for the production of polyunsaturated fatty acid fortified eggs (NY/T 4070-2021). In addition to their practical significance, such implementation will attract considerable interest in the study of consumers’ preference for nutritionally fortified eggs and their response to Supplementary Information about their health benefits. Widespread consumer acceptance of nutritionally fortified eggs will significantly increase the capacity of egg processing enterprises’ production technology and improve product market competitiveness.

Studies have shown that increasing numbers of stakeholders are embracing alternative approaches, which are softer and involve mostly educational and industry-related voluntary codes based on behavioural economic priciples, to alter choice environments and improve consumer choices, such as nudging [27,28]. By providing more external scientific knowledge and international market information, and grasping consumers’ response to the stimulation of nutritional quality information, not only will food enterprises benefit by optimizing their marketing strategies, but the empirical support will also enable the government to formulate nutrition fortified food standards, disseminate nutrition education, and improve information supply to overcome market failures [29].

In this study, we employed a choice experiment (CE) survey to investigate consumers’ preferences and willingness to pay (WTP) for eggs with different attributes by explicitly considering the effects of information treatment. In particular, consumers were provided with information regarding the functional eggs’ nutritional benefits, as well as data on their current market status in developed countries. The main aims of this study were, therefore, threefold: (1) to analyze consumer preferences for the nutrient contents, organic and free-range production methods, and brand-related attributes of eggs, thus determining the part-worth utility values that each of these attributes provide, especially in terms of the interactions between nutritional enrichment and other attributes; (2) to gain insight into the potential impacts of information treatment on consumers’ preferences and WTP for functional eggs; and (3) to explore the heterogeneous effects of information treatment according to different consumers’ segmentations.

## 2. Method

### 2.1. Theoretical Analysis of Consumers’ Preference

The theoretical basis of this study is the random utility theory [30,31]. Consumer preference is conveyed via bundles of commodity characteristics, with each product described by a group of attributes with different levels. Based on the random utility theory, the choice experiment method was applied to set the attribute combination level of the research object and then to form different selection sets.

Let $U_{ijn}$ represent consumer i’s utility by choosing the j–th product in the n–th choice question. As described previously by Hu, Batte, Woods and Ernst [11] and Ubilava and Foster [32], we assume a linear presentation of the utility as a function of product attributes $X_{ijn}$, and thus have:

(1) $$U_{ijn}=\alpha X_{ijn}+\varepsilon_{ijn}$$

where a is a vector of unknown part-worth utilities that is associated with product attributes, and $\varepsilon_{ijn}$ is the independently identically distributed random component of utility function. According to the random utility theory, respondents choose a product in a specific choice question only when this alternative provides the largest utility compared to the other options offered in that question.

### 2.2. Choice Experiment Design

To estimate consumers’ preference for egg characteristics, a CE was applied in which participants were requested to make repeated choices between three eggs and a “none of the above” option. The application of these CE questions may be likened to the consumers’ decision-making processes while shopping [33].

Five egg attributes were selected in our choice experiment, namely nutrition (nutritionally enriched or normal), organic certification, the rearing conditions of hens (free-range or not^1^), brand (habitual purchase brands or non-habitual purchase brands), and price (Table 1). These attributes were chosen based on the results of previous in-store surveys, both in China [34,35] and around the world [4,5,33,36,37], which showed that organic certification, animal welfare, brand, and price were among the most important characteristics in egg purchase decisions. Given the focus of this study, the claim of nutritional fortification and price were prerequisites; however, it was also important to place these two attributes within the context of other egg attributes that also influence consumers’ choice, in order to describe an egg that people can see and purchase anywhere in their daily life in China. There is, in general, little published data on consumers’ reactions to a combination of functional food attributes that include organic, rearing conditions (production method), and brand [4,5,38], and no studies involve Chinese consumers.

Currently, omega-3-enriched eggs, selenium-enriched eggs, and folic acid-enriched eggs are those most commonly available in China’s market. Our research was, thus, limited to these three kinds of eggs, which were collectively referred to as nutritionally fortified eggs in our survey. Furthermore, the decision to include organic certification and rearing conditions as attributes was driven by the growing concern for food products with respect to food safety, taste, and animal welfare principles both in China [39] and internationally [37,40,41]. Since Chinese consumers do not habitually purchase functional eggs, the survey further divided the attributes of egg brands into “habitually purchased brands”, with which participants were familiar and purchased regularly, and “non-habitually purchased brands”. Consumers are known to have a certain degree of trust in and loyalty towards familiar brands that they purchase frequently, and these brands could be therefore considered to be a search attribute, the impact of which is similar to a quality information label [42]. In addition, to determine the optimal price range for nutritionally fortified eggs, Chinese market research was carried out before the formal survey design. Consequently, our experiment set the prices ranging from 1 CNY per egg to 4 CNY per egg, in 1 CNY increments, and thus provided four levels for the attribute of price. As an attribute, price helps to generate part-worth to estimate the monetary value that consumers assign to the presence of the other attributes, thus providing certain implications for related market stakeholders.

**Figure 1:**
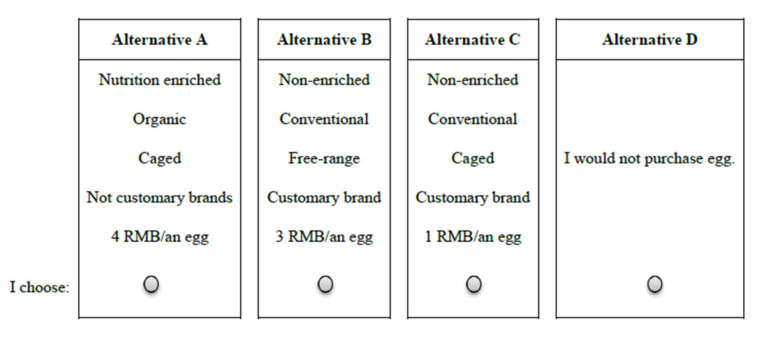
Example of a choice set used in the choice experiment questions.

An orthogonal factorial design approach was applied to the survey by generating 16 choice sets of 48 possible alternatives^3^ using SAS software [43]. Each participant was required to answer five discrete choice questions randomly among these 16 choice tasks regarding which egg they would be likely to buy. Each question provided three alternative egg products, as well as a “no purchase” option. An example of a choice task is presented in Figure 1. The choice tasks and alternatives in each question were randomized to reduce the order effect [44].

### 2.3. Information Treatments Design

In our survey, respondents were randomly assigned to either the information treatment group or control group. In the control group, respondents were not offered any additional information and, consequently, utilized whatever perceptions they had before the survey, just as they would when entering a store to buy eggs in daily life.

**Figure 2:**
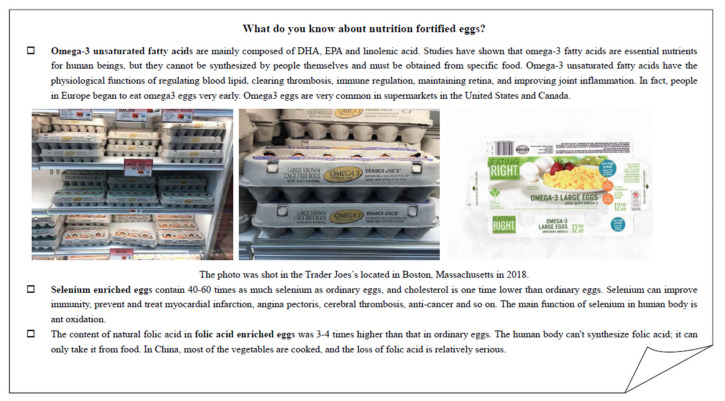
Information card shown to the treatment group.

The information treatment content was comprised of two parts. The first part included scientific information and health outcomes of two trace elements (selenium and the folic acids) and unsaturated fatty acids, since a lack of knowledge about the benefits related to the consumption of a functional ingredient could discourage purchase intention. The second part of the information treatment introduced the status of functional eggs in the markets of the United States of America and Canada. Table S1 in the Supplementary Materials presents an English translation of the information treatment text in detail, as presented to respondents in the treatment group in our survey. The computer system randomly presented participants with an information card, as shown in Figure 2. Respondents had at least one minute to read the text and could request to re-read the information at any time while answering the choice experiment questions. Respondents could not skip the information card within one minute.

As in a previous study exploring the role of health claim on consumers’ preference [10,45,46], we apply the between-subjects experiment to assess the effect of information treatment. The reasons for applying a between-subjects instead of a within-subjects experiment is because they are mostly preferred compared to compared to within-subject designs in behavioral economics, and most importantly because between-subjects experiments would better mimic a real situation that some consumers may find a health claim on the package while some might not be aware of the information.

### 2.4. Econometric Specification and Estimation

Since we assumed that the respondents would have different preferences for each attribute, the mixed logit (ML) model, one of the most widely applied approaches for regression, was applied. The ML model reveals the unobserved heterogeneity in consumer choices through a general specification of the part-worth utilities defined on the whole sample [11]. Then, the choice probability is:

(2) $$\mathrm{Prob}\left(Y_{in}=j\right)=\int \frac{\exp\left(X_{ijn}\right)}{\sum_{j=1}^{4}X_{ijn}}f\left(\alpha \right)d\alpha$$

In our model, we initially hypothesized that all of the parameters were modeled as random, including all interaction terms between attributes [22,47]; however, some papers regard the parameters on price variables and/or interaction terms as fixed [44,48]. Therefore, this study also conducted a simulation in which all parameters were treated as random with the exception of price and interactions, but no changes were found in the sign of coefficients. The results are shown in Table S2 in the Supplementary Materials.

We also assumed that all of the parameters, except for the price coefficient, were normally distributed, since there was no theoretical foundation or evidence for whether these attributes should positively or negatively affect consumers’ preference and utilities a priori, based on existing literature.

Based on the coefficient estimation, we could obtain respondents’ WTP as follows:

(3) $${\mathrm{WTP}}_{k}=-\frac{\alpha_{k}+\sum_{m}\alpha_{k\times m}}{\alpha_{\mathrm{price}}}$$

where $\alpha_{k}$ represents the coefficient for the attributes, $\alpha_{\mathrm{price}}$ is the estimated price coefficient, and $\alpha_{k\times m}$ is the coefficient of the m-th level of k-th attribute interacting with other attributes or variables.

In the process of empirical regression, we set information treatment as a dummy variable, which equals 1 if a respondent read the information card and 0 otherwise. The information treatment variable and the nutrition enrichment as the interaction item were set to estimate WTP in the pooled sample. The pooled sample was then further divided into a treatment group and a control group. For each group, we estimated individual-level WTP and performed pairwise comparisons on the means of WTP between the two groups. Wilcoxon–Mann–Whitney tests were conducted to assess the distributions of the treatment group and the control group using Stata’s rank sum test. The results of all test statistics were found to be insignificant, at 10%, indicating that null hypotheses could not be rejected.

## 3. Experiment Implementation

**Figure 3:**
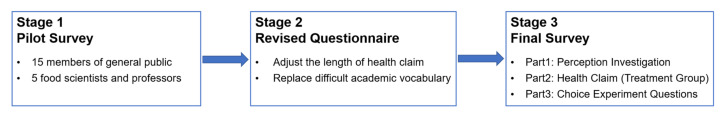
Different stages of research process.

In this study, we cooperated with V-insight Market Research Inc. (Beijing, China), a third-party contractor based in Beijing, and utilized its consumer panel database to obtain our sample. Figure 3 is a graphical presentation of the different stages of this study with the order in which they are carried out. We first solicited feedback and suggestions about the clarity and accuracy of our draft questionnaires from 15 members of the general public as well as five subject matter experts and professors in the field. The purpose of this process was to acquaint ourselves with the expected time commitment for participants to complete the survey and to guarantee that all the professional terms in both the information treatment and questions were correct and easy to understand. This pilot survey was particularly useful, because the original intention was to have a two-page information intervention. Most of the respondents found such an information card was too long, was difficult to understand within limited time, and, consequently, lost their patience after reading too many words at the beginning of the survey. Instead, we compressed the information treatment into half a page and replaced a large number of difficult academic words with more colloquial expressions.

The questionnaire comprised three main sections. First, the survey collected information about the possible influencing factors on consumer preferences, including demographic information, the individual’s egg consumption habits (including which egg types they usually purchase and purchase frequency of different eggs), their knowledge of unsaturated fatty acids, and nutritional knowledge acquisition channels^4^. Second, some participants were randomly involved in the information treatment. Third, the CE questions were applied to all participants.

This survey was conducted in Beijing, Shanghai, Nanjing, and Xi’an in 2020. The respondents were all consumers who had bought eggs within the previous six months. All respondents were aged above 18 and were responsible for food consumption within their household or, at least, participated in food consumption decisions in their daily lives. The demographic characteristics of the sample presented in summary in Table 2. There were 740 participants, of which 364 people received additional information (treatment group), accounting for 49.19% of the total sample; the remaining 376 received no information treatment and were regarded as the control group.

## 4. Empirical Results and Discussion

### 4.1. Consumers’ Perception and Purchase Frequency of Nutritional-Enriched Eggs

Table 3 shows respondents’ familiarity and purchase frequency of the three nutritionally enriched eggs. The results indicated that 313 respondents had previously heard about selenium-enriched eggs, suggesting that consumers had higher recognition of selenium-enriched eggs than the other nutritionally fortified eggs. A total of 87% of respondents had purchased free-range eggs, 56% had purchased organic eggs, while only approximately 34% of the sample had purchased one or more types of nutritionally fortified eggs. The results show that compared with organic or free-range eggs, consumers have low acceptance of nutritious eggs and lack of purchase experience.

A large majority of the respondents who had heard of omega-3 eggs, but had not bought them, expressed that they were not familiar with the nutrition content, efficacy, or the production process involved in omega-3 eggs. Many respondents agreed that omega-3 eggs might be good for people’s health; however, they had poor knowledge about the health benefits and were concerned about false media campaigns and advertising. Some participants said they did not know where to buy omega-3 eggs and that very few people they knew had purchased them.

While some respondents stated that they believed nutritionally fortified eggs to be a healthy food, there was a wide gap in their perceptions of the health benefits of nutritious content, particularly with regard to unsaturated fatty acids. For example, only 57% of respondents claimed to have even a rudimentary understanding of omega-3 fatty acids. However, when our study investigated the intention of consumers to purchase nutritionally fortified eggs, the results surprisingly showed that 78% of respondents expressed their strong interest and willingness to do so, despite their lack of a comprehensive understanding of different trace elements.

**Figure 4:**
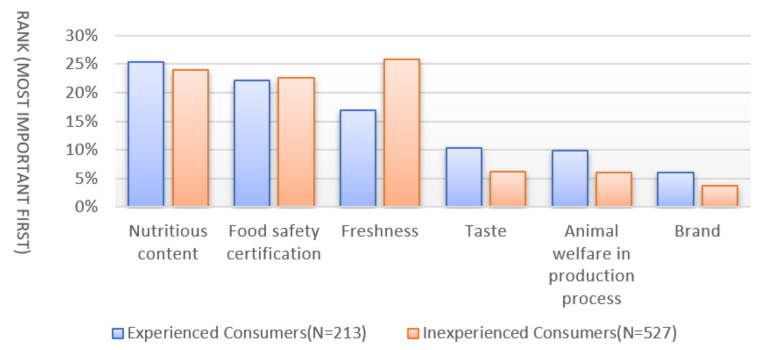
Consumers perceived importance of egg characteristics.

The survey also attempted to ascertain why the information provided had different impacts on consumers with purchasing experience^2^ of nutritionally enriched eggs and investigated how experienced and inexperienced consumers respond differently in terms of their prior perceptions for all attributes. Participants were asked to choose the one factor out of 15 potential factors that they perceived to be most important in their egg buying decisions. The ranks and percentages of the top six factors selected by both experienced and inexperienced consumer participants are presented in Figure 4. In general, the three main factors considered when buying eggs are nutrition content, freshness, and safety certification. More experienced consumers (25.35%) than inexperienced consumers (23.91%) selected the nutrition content as one of the top factors of concern. This result indicated that experienced consumers perceived higher level of importance on nutritional quality in the egg production practice than inexperienced consumers. The different perceptions could serve as potential explanations for the heterogeneous informational effects.

### 4.2. Consumer Preference Estimations

The summarized results for the pooled samples in the treatment group and control group are presented in Table 4. Our results show that the provision of information about active ingredients, effective function, health outcomes of the two trace elements and the unsaturated fatty acids, and current global market situations contribute significantly to the establishment of Chinese consumers’ preferences for certain eggs. The provision of information led to increased part-worth utilities, thereby increasing the possibility of selection for functional eggs. The participating consumers in our study revealed preferences for nutrition-related claims with additional information on health outcomes and the market status quo, thus confirming the previous studies of Żakowska-Biemans and Tekień [5], Kleef, Trijpa and Luning [15], and Yeh, Menozzi and Török [25].

The regression results from the pooled sample show that the coefficient of “enriched x info” was significantly positive, indicating that consumers who received additional information were more likely to buy nutrition enriched eggs than normal eggs. We found that nutrition enrichment had a significant positive coefficient in the control group, indicating that consumers retained their positive preference for nutrition enriched eggs regardless of whether or not they received additional information. However, the coefficient of the treatment group was larger than that of the control group, indicating that the consumers who received additional information had a higher utility level for nutritional fortification, and were, thus, more likely to choose nutrition fortified eggs.

The coefficients of organic certification and free-range production were both positive and significant, indicating that consumers prefer to buy eggs with organic certification and free-range labels than normal or caged-hen eggs. Indeed, previous studies have found consumers to place a higher part-worth utility on eggs produced in organic and cage-free systems because these are perceived to have been produced with higher standards of production and animal welfare [5,12,36,49,50,51,52]. In our survey, more than 96% of respondents asserted their belief that organic certification is a significant guarantee of food safety and enhanced nutrition.

The significant positive effect of a familiar brand showed that consumers were more likely to buy egg brands with which they were familiar and purchased regularly. This is consistent with the research results reported by Annunziata and Vecchio [14], Brakus*,* et al. [53], and Jensen and Hansen [54]. It is widely accepted that consumers tend to accumulate trust in a specific brand after purchasing it regularly over a long time.

The factor of price was found to be significantly negative, indicating that an increase in the cost of eggs would lead to a decrease in consumer utility, thus reducing the probability of purchase. The coefficient of the variable “no purchase” was found to be negative, indicating that respondents generally preferred to select one of the egg alternatives in the CE tasks and avoided the option of “I would not buy eggs”.

The interaction term between nutrition enrichment and organic was found to be negative, revealing a substitution relationship between them. Our survey results showed that most Chinese consumers currently believe that an organic certification indicates a guarantee of nutritional quality and production safety; therefore, claims of nutritional fortification may have a repetitive effect on people’s intention to purchase. Furthermore, more than 93% of respondents asserted the belief that the additional nutritional components in functional eggs come mainly from nutritional fortifiers, and only 4% of them displayed a comprehensive understanding of feed composition during the egg production process. Considering that consumers who prefer to buy organic eggs may believe that organic certification ensures a more natural animal feed, it follows that they may also be sensitive to labels of artificial food additives and experience a sense of conflict when faced simultaneously with labels organic and nutrition enhancement attributes. This concurs with the findings of Yeh, Menozzi and Török [25] that the combination of health- and nutrition-related claims did not significantly improve people’s preference for organic eggs, because the more expensive price of organic products was a more significant barrier for consumers in their purchase decisions.

Furthermore, the interaction coefficient between nutritional fortification and familiar brand was surprisingly found to be negative in this study, indicating that consumers have some doubts about the nutritional fortification claimed by a brand, even one that is familiar and frequently purchased. One possible reason for this result is that Chinese consumers are concerned about dietary nutrition and therefore show great interest and potential purchase intention in nutritionally enriched eggs. However, since nutritionally enriched eggs are not commonly distributed or sold on the Chinese market, consumers remain doubtful about claims made by producers alone. Thus, the combined attributes of nutrient enrichment and trusted brand might not increase consumers’ possibility of purchase. When asked which channels the respondents in our survey would prefer to obtain information and/or claims of certification about functional eggs, only 45% indicated that advertisements from familiar brands would be considered a reliable source of nutrition information.

The interaction term of organic and free-range was found to be negative, indicating a substitution relationship between them [47]. It was found that 57% of respondents did not have a distinct preference between organic and free-range when buying eggs, and 72% of respondents did not have a comprehensive understanding of the different standards of organic eggs. This result corroborates findings from previous works. Żakowska-Biemans and Tekień [5] found that Polish consumers considered free range and organic claims to be competing concepts, mainly because organic production is perceived to satisfy the same outdoor requirements as free-range eggs despite the fact that the production of organic eggs is governed by much stricter standards than that of free-range eggs. Similarly, Heng and Peterson [55] found that consumers in their sample did not value the combination of a cage-free label and organic certification, since organic eggs are naturally cage-free, and nearly 40% of the respondents in their sample stated that cage-free is a somewhat important or extremely important factor in their choice of organic eggs.

### 4.3. Information Impact on Consumers’ Willingness-to-Pay

The WTP estimation results for the attributes are presented in Table 5. Respondents who received additional information were willing to pay 4.65 CNY more for nutritionally fortified eggs than for ordinary eggs, while consumers without the information treatment would like to pay 2.55 CNY more to buy nutritionally enriched eggs. The provision of additional health benefits and market-related information helped to increase people’s WTP by 2.10 CNY in our experiment, proving that information treatment could effectively improve consumers’ purchase confidence and their WTP. It is worth noting that the consumers’ WTP for nutritionally fortified eggs in the control group was positive, and even higher than for the other four attributes, indicating that even without information intervention, consumers’ WTP for nutritionally fortified eggs was high. The information stimulation enhanced consumers’ WTP by strengthening their existing preference for nutritionally fortified eggs.

Several distinct consumer segments are reported to significantly affect respondents’ perceptions, preferences, behaviors, and WTP for eggs [12]. The most common techniques for segmenting consumers are via sociodemographics, trust, or on the basis of stated purchase behaviors [36,56,57]. Further to the findings in the research literature, we divided the samples in this study into four different categories and obtained eight dummy variables, according to participants’ family structure, trust in nutrient contents, purchasing experience, and household income. We then used this model to interact the eight dummy variables with the attributes of nutritional fortification to estimate the WTP values (Table 6). Overall, participants who received additional information about the value of nutrients and the market status quo were willing to pay more for the nutritionally fortified eggs, regardless of their family composition, subjective stated trust, prior consumption experience or household income.

Specifically, respondents with children were likely to pay the most among the subsamples, regardless of information treatment. This may be due to the fact that although consumers with children might have a low grade of familiarity with functional eggs, they are likely to be more sensitive to any nutritional information about maternal and infant health, especially when advised that folic acid and unsaturated fatty acids are conducive to the growth of infants and young children. In our survey, 86% of the respondents had children at home and, according to their responses to an open question about daily food consumption behaviors, preferred to procure foods with a high nutritional value.

Survey respondents who expressed doubt as to the effectivity of the nutritional elements in nutritionally fortified eggs on human health were found to have the lowest WTP for nutritional enrichment. However, the provision of additional information helped to increase their WTP from 0.06 CNY to 2.01 CNY. In contrast, WTP by those who initially expressed the belief that functional eggs have certain health benefits was seen to increase by only 0.50 CNY after the provision of additional information. This result implies that information treatment may have a greater marginal effect on those who initially suspicious about the nutritional fortification of eggs than that for those who are already convinced about the efficacy of the additional nutrients.

Previous purchasing experience plays an important role in consumers’ decision making [5,36,50]. The WTP premium of experienced consumers is 1.5 times that of inexperienced consumers without any additional information, indicating that purchasing experience will build their confidence on continuously making a consumption decision. The result also reflects that information treatment is more effective to improve the WTP of experienced consumers than in inexperienced consumers. It further indicates that experienced consumers are more likely to value nutritional enrichment and, thus, may be more sensitive to information about health benefits and the market status quo.

Additionally, it was found that consumers with lower annual household incomes were only willing to spend a little extra (0.77 CNY) for nutritionally fortified eggs than for ordinary eggs, which is less than those respondents with a relatively high income (1.69 CNY) in the control group. However, the information had a larger effect on the low-income group than on the high-income group. The treatment increased average respondents’ WTP of the lower income group by about 2.13 CNY, while increased about 1.51 CNY in the higher income group. It shows that although the respondents with lower income are not willing to pay too much money for nutritional fortification attributes, they are more sensitive to external information than those with higher income. In fact, nutritionally fortified eggs are still more expensive than ordinary eggs in the Chinese market. For lower income groups, price is still a factor limiting their long-term and stable purchase experience, and does not generate a certain understanding of the health benefits of nutritionally fortified eggs. Therefore, for such consumers, when we give additional information, they will have a greater marginal response.

## 5. Conclusions

The study of the effects of nutrition and health information treatments on consumer preference is important and rewarding as it contributes to the promotion of healthier diets and a sustainable food system in China; therefore, it is of both theoretical value and practical significance.

The findings of our study suggest that, although many believe that the consumption of nutritionally fortified eggs is healthy, there remains a considerable lack of knowledge regarding the details of their health benefits. Consumers without relevant knowledge rely on external information to reduce uncertainty and risks when making the decision to purchase nutritionally fortified eggs. The provision of information about the health outcomes and current market status quo could therefore effectively improve the part-worth utility, choice probability, and WTP for nutritionally fortified eggs.

Furthermore, information treatment is impacted by obvious heterogeneity in its promotion of consumer preference. More specifically, survey respondents with children were found to be more sensitive to information treatment about the health benefits of functional eggs. Findings in this study also suggest that information that aims to educate people can have higher marginal effects on experienced consumers than on inexperienced consumers. Consumers who expressed their trust in the nutritional and health-related value of functional eggs before the information treatment did not respond more actively after the treatment. Those with a relatively low household income indicated a small WTP, but tended to respond more strongly to the information treatment than the higher income group.

The results showed that the attributes of nutritional enrichment and organic certification might be perceived to be competing concepts, referring to a more healthy and nutritious choice of eggs. This implies that if production and sales enterprises provide more detailed information about nutritional fortification technology and the egg production process, it will help consumers to effectively distinguish the relationship between nutritional fortification and organic production and may therefore have an effective impact on consumers’ cognition and purchase intentions.

The findings of our study suggest that functional eggs have a larger premium space than ordinary eggs in China. Hence, on the premise of further improving the income of Chinese residents and weakening the problem of information asymmetry in the nutritionally fortified egg market, it is evident that eggs with a higher nutritional quality could potentially realize high quality and good prices. Therefore, the government could accelerate the promotion of nutrition fortification standards, could standardize guidelines for the use of nutritional fortification labels and statements, and prevent enterprises from distributing misleading or false publicity on the nutritional fortification attributes that concern consumers. In addition, government and scientific research institutions could increase efforts to popularize knowledge relating to trace elements and unsaturated fatty acids, to improve consumer acceptance, purchase confidence, and WTP for nutritionally fortified foods. In view of the heterogeneity of consumption preferences, enterprises could accurately identify market segments and implement differentiated marketing strategies. This strategic intervention would highlight the nutritional content and health value of nutrient-fortified eggs, and, at the same time, popularize relevant information about the production process and nutritional fortification technology involved in nutritionally fortified eggs.

1. Free-range eggs are popular in China, mainly because they are perceived to be more healthful, nutritious, and tasty than caged eggs, since the hens are not reared in cages but have access to run freely outdoors and benefit from multiple natural factors. Moreover, as Chinese consumers are increasingly concerned about improving animal welfare, they are increasingly inclined to purchase free-range eggs.
2. Based on the self-reported purchase experiences in this study, experienced consumers were defined as those who had purchased either selenium-enriched, omega-3-enriched, or folic acid-enriched eggs within the previous six-month period. In contrast, inexperienced consumers were those who had never purchased any of the three functional eggs in our survey.
3. Five attributes were used to characterize an egg product in the choice experiment, yielding 2 × 2 × 2 × 2 × 4 = 64 possible alternatives in total. However, as it was considered overwhelming for participants to answer a large number of repeated CE questions in the survey, the SAS was introduced to create 48 alternatives to generate 16 CE questions based on the D-efficiency criterion, a standard method of quantifying the relative efficiency of particular experiment design [48]. Each question had three response options as well as an “I would not purchase eggs” option.
4. The first part of our questionnaire investigated consumers’ demographic information and perception. To begin with, respondents provide their city of residence, gender, age, education level, household income, marriage, and family status. Then, respondents were asked directly whether they fully understand what functional eggs are and whether they had purchased them over the preceding six months. Meanwhile, an open-ended question required respondents to state the reasons why they had not previously purchased nutritionally enriched eggs although they had heard them before. We asked consumers to choose multiple factors they would consider when buying eggs from 16 options, and choose one of the most important factors that affect their choice at the same time. Consumers were then asked whether they understand the relationship between unsaturated fatty acids, folic acid, selenium, and human health successively. All these three questions were rated on a five-point Likert scale ranging from “not at all” (1) to “fully aware of” (5). In addition, we provide respondents with existing media channels to obtain nutrition and health information, so that respondents can choose from which channels they want to obtain information about functional eggs.

## Figures and Tables

**Table 1:** Attributes and levels used in the choice design.

Attributes	Levels	Description
Nutrition Enrichment	Enriched	Refers to whether the egg is enriched with omega-3, selenium, or folic acid.
	Normal *	
Organic Certification	Organic	Refers to whether the egg has an organic certification on the package.
	Conventional *	
Rearing Conditions	Free-range	Refers to whether the egg is caged-free or not.
	Caged *	
Brand	Habitual purchase brands	Refers to whether it is a brand that consumers are familiar with and often buy.
	Not habitual purchase brands *	
Price	1 CNY	Refers to price for per egg in the market where the respondents typically shop.
	2 CNY	
	3 CNY	
	4 CNY	

Note: * represents the base level.

**Table 2:** Characteristics of survey sample.

Variables		Pooled Sample	Proportion (%)	Treatment Group
Gender	Male	222	30.00	107
	Female	518	70.00	257
Education	High school	69	9.32	32
	Bachelor’s degree	627	84.73	313
	Graduate degree or above	44	5.95	19
Age	25–29	165	22.30	78
	30–39	165	22.30	79
	40–49	205	27.70	102
	50 and above	205	27.70	105
Cities	Beijing	428	57.84	201
	Shanghai	104	14.05	51
	Nanjing	104	14.05	54
	Xi’an	104	14.05	58
Household size and marital status	Single	49	6.62	26
	Married without children	48	6.49	25
	Married and have children	640	86.49	313
	Others	3	0.41	0
Annual household income	Below 100,000 CNY	18	2.43	6
	100,000–150,000 CNY	71	9.59	38
	160,000–200,000 CNY	118	15.95	54
	210,000–300,000 CNY	229	30.95	121
	310,000–400,000 CNY	163	22.03	81
	410,000–500,000 CNY	74	10.00	36
	510,000–700,000 CNY	33	4.46	14
	700,000–1,000,000 CNY	19	2.57	6
	1,000,000+ CNY	15	2.03	8

Note: Proportion was summarized based on the pooled sample. For example, 30% of the total 740 participants were male in the research sample, and 107 of them were in the treatment group. Females accounted for 70% of the pool sample, a much higher representation than males. Most respondents were 40 years or older. The proportion of married people with children was 86.49%, which could better reflect the demand for eggs of people who usually eat at home.

**Table 3:** Participant’s cognition and purchase experience of the nutrition fortified eggs.

Statements	Selenium-Enriched Eggs	Omega-3-Enriched Eggs	Folic Acid-Enriched Eggs
I have heard about … before.	313	86	103
	(42.30%)	(11.62%)	(13.92%)
I bought … in recent 6 months.	176	20	53
	(23.78%)	(2.70%)	(7.16%)

Note: Percentages were summarized for the pool sample including both control and treatment group, shown in the brackets. Some participants bought more than one type of nutrition-fortified eggs.

**Table 4:** Estimation results using a mixed logit model.

Variables	Pooled Sample	Treatment Group	Control Group
Main effects			
Enriched	0.811 ***	1.426 ***	1.090 ***
	(0.147)	(0.456)	(0.324)
Organic	0.699 ***	0.948 ***	0.869 ***
	(0.158)	(0.299)	(0.275)
Free-range	0.488 ***	0.664 ***	0.659 ***
	(0.120)	(0.237)	(0.231)
Habitual purchase brands	0.227 ***	0.251 **	0.331 ***
	(0.060)	(0.106)	(0.125)
Price	−0.264 ***	−0.307 **	−0.428 ***
	(0.065)	(0.130)	(0.165)
No purchase	−1.034 ***	−3.505 ***	−0.984 ***
	(0.211)	(1.160)	(0.324)
Interactive effects			
Enriched × Info	0.245 ***		
	(0.095)		
Enriched × Organic	−0.205 ***		
	(0.048)		
Enriched × Free-range	−0.042		
	(0.082)		
Enriched × Habitual purchase brands	−0.140 **		
	(0.064)		
Organic × Free-range	−0.136 **		
	(0.056)		
Organic × Habitual purchase brands	−0.071 *		
	(0.043)		
Free-range × Habitual purchase brands	0.144 ***		
	(0.047)		
Log likelihood	−3997.7100	−1897.3239	−2082.9490
Observations	14800	7280	7520

Note: ***, **, and * indicate significance at the 1%, 5%, and 10% levels, respectively. All standard errors are in parentheses. Observations (14,800) = 740 participants × 5 question × 4 alternatives. For brevity, we did not report the estimates of the standard deviations of the random parameters. All standard deviations are statistically significant at the 5% significance level, indicating heterogeneous preference. All parameters were modeled as random parameters in this model.

**Table 5:** Estimated willingness-to-pay (CNY per egg) for different attributes.

Attributes	(1)	(2)	(3)
	Pooled Samples	Treatment Group	Control Group
Enriched	3.08	4.65	2.55
	(2.30, 3.85)	(2.50, 6.79)	(1.82, 3.27)
Organic	2.65	3.09	2.03
	(1.90, 3.41)	(1.53, 4.65)	(1.39, 2.67)
Free-range	1.85	2.16	1.54
	(1.34, 2.37)	(1.11, 3.21)	(1.04, 2.04)
Habitual purchase brands	0.86	0.82	0.77
	(0.47, 1.25)	(0.17, 1.47)	(0.37, 1.17)
No purchase	–3.92	–11.43	–2.30
	(–6.28, –1.57)	(–19.26, –3.59)	(–4.05, 0.55)

Note: The WTP estimates were calculated from the results from Table 4. Top lines give estimated mean WTP value, and 95% confidence intervals (calculated using the Krinsky–Robb procedure) are in parentheses.

**Table 6:** Heterogeneity of consumers’ WTP (CNY per egg).

Subsamples	Treatment Group	Control Group	Difference
With children	7.87	4.32	3.55
	(0.71, 15.03)	(2.13, 6.52)	
Without children	3.72	0.81	2.91
	(–0.53, 7.96)	(0.36, 1.26)	
Trust ^†^	2.78	2.28	0.50
	(–0.02, 5.58)	(–0.59, 5.15)	
Distrust ^‡^	2.01	0.06	1.95
	(0.35, 3.67)	(–1.21, 1.33)	
Experienced consumer ^§^	7.40	1.96	5.44
	(0.45, 14.35)	(0.91, 3.00)	
Inexperienced consumer ^¶^	6.72	1.70	5.02
	(–1.66, 15.09)	(1.24, 2.16)	
Household annual income at least 300,000 CNY	3.19	1.69	1.51
	(1.84, 4.55)	(1.26, 2.12)	
Household annual income less than 300,000 CNY	2.90	0.77	2.13
	(0.06, 5.75)	(−0.17, 1.72)	

Note: Top lines give estimated mean WTP value, and 95% confidence intervals (calculated using the Krinsky–Robb procedure) are in parentheses. The column 4 “information effect” is the difference between the WTP value of treatment (column 2) and control group (column 3) for each subgroup respectively. ^†^ Trust is a dummy variable, which equals to 1 if participants stated that they would love to purchase nutrition fortified eggs because they trust the nutrient contents have health benefits to human being, and 0 if they were reluctant to try because of distrust. ^‡^ Distrust is a dummy variable, which equals to 1 if participants chose that they do not trust the nutrient contents have health benefits to human being and thus they do not want to buy any nutritionally fortified eggs, but is 0 otherwise. ^§^ Experienced consumer is a dummy variable, which equal to 1 if a participant reported to buy nutritionally fortified eggs in the last 6 months, and 0 otherwise. ^¶^ Inexperienced consumer is a dummy variable, which equal to 1 if a participant reported has never bought nutritional fortified eggs before, and 0 otherwise.

## Data Availability

The data presented in this study are available on request from the corresponding author. The data are not publicly available now due to the Annual Project of the National Social Science Fund of China [grant number: 21BGL247] is not yet closed.

## Supplementary Materials

The following are available online at https://www.mdpi.com/article/10.3390/foods11081145/s1, Table S1: Content of information treatment translated from Chinese, Table S2: Estimation results using different random parameters.
