# Supplementary material for: Consumer Preference for Nutritionally Fortified Eggs and Impact of Health Benefit Information
Source: Foods. 2022 Apr 15;11(8):1145. doi: 10.3390/foods11081145 (PMC9025974; doi:10.3390/foods11081145)
Supplement: Supplementary file 1 [file foods-11-01145-s001.zip › foods-1654400-supplementary.pdf]

Supplementary Materials

Table S1 Content of information treatment translated from Chinese

- ☐ **Omega-3 unsaturated fatty acids** are mainly composed of DHA, EPA, and linolenic acid. Studies have shown that omega-3 fatty acids are essential nutrients for human beings, but they cannot be synthesized by people themselves and must be obtained from a specific food. Omega-3 unsaturated fatty acids have the physiological functions of regulating blood lipid, clearing thrombosis, immune regulation, maintaining retina, and improving joint inflammation. Omega3 eggs are very common in supermarkets in the United States and Canada (Please refer to the photo which was taken in the local market).
- ☐ **Selenium-enriched eggs** contain 40-60 times as much selenium as ordinary eggs, and cholesterol is one time lower than ordinary eggs. Selenium can improve immunity, prevent and treat myocardial infarction, angina pectoris, cerebral thrombosis, and helps to fight against cancers. The basic function of selenium in the human body is ant oxidation.
- ☐ The content of natural folic acid in **folic acid-enriched eggs** was 3-4 times higher than that in ordinary eggs. The human body can't synthesize folic acid; it can only take it from foods. In China, most of the vegetables are fully cooked, and the loss of folic acid is relatively serious.
- ☐ All the information above is based on the interview with the experts from the Institute of Food and Nutrition Development, the Ministry of Agriculture and Rural Affairs of the People’s Republic of China.

Table S2 Estimation results using different random parameters

Variables	Pooled Sample	Treatment Group	Control Group
Main Effects			
Enriched	0.592***	0.703***	0.681***
	(0.054)	(0.071)	(0.075)
Organic	0.511***	0.490***	0.539***
	(0.047)	(0.058)	(0.069)
Free-range	0.354***	0.312***	0.394***
	(0.052)	(0.067)	(0.074)
Customary brand	0.169***	0.144***	0.192***
	(0.035)	(0.049)	(0.049)
Price	-0.183***	-0.131***	-0.243***
	(0.035)	(0.044)	(0.053)
No purchase	-0.947***	-1.102***	-0.791***
	(0.212)	(0.264)	(0.258)
Interactive effects			
Enriched x Info	0.181***		
	(0.051)		
Enriched x Organic	-0.154***	-0.152***	-0.177***
	(0.031)	(0.042)	(0.046)
Enriched x Free-range	-0.039	-0.037	-0.062
	(0.045)	(0.055)	(0.061)
Enriched x Customary brand	-0.113**	-0.067	-0.137**
	(0.048)	(0.056)	(0.057)
Organic x Free-range	-0.078**	-0.030	-0.124***
	(0.031)	(0.044)	(0.046)
Organic x Customary brand	-0.036	0.021	-0.096*
	(0.034)	(0.043)	(0.050)
Free-range x Customary brand	0.103***	0.094*	0.124**
	(0.037)	(0.048)	(0.052)
Log likelihood	-4009.5687	-1912.1524	-2089.894
Observations	14800	7280	7520

Note: ***, **, and * indicate significance at the 1%, 5%, and 10% levels respectively. All standards errors are in parentheses. For brevity, we did not report the estimates of the standard deviations of the random parameters. The parameters for price and interaction terms were specified to be fixed, and the other parameters were random.
